# Editorial: Synergistic combinatorial treatments to overcome antibiotic resistance

**DOI:** 10.3389/fcimb.2024.1369264

**Published:** 2024-01-26

**Authors:** Javier A. Garza-Cervantes, Angel León-Buitimea

**Affiliations:** ^1^ Facultad de Ciencias Químicas, Universidad Autónoma de Nuevo León (UANL), San Nicolás de los Garza, NL, Mexico; ^2^ Centro de Investigación en Biotecnología y Nanotecnología, Facultad de Ciencias Químicas, Universidad Autónoma de Nuevo León, Parque de Investigación e Innovación Tecnológica, Apodaca, Nuevo León, Mexico; ^3^ Tecnologico de Monterrey, School of Engineering and Sciences, Monterrey, Mexico

**Keywords:** antibiotic resistance, synergistic treatment, antimicrobial nanocomposites, green synthesis, antimicrobial peptides, antimicrobial biopolymers, antimicrobial nanoparticles

Antimicrobial resistance (AMR) looms as a growing threat, with “superbugs”(Salam et al., 2023) like the WHO ESKAPE pathogens (*Enterococcus faecium*, *Staphylococcus aureus*, *Klebsiella pneumoniae*, *Acinetobacter baumannii*, *Pseudomonas aeruginosa*, and *Enterobacter* spp.) exhibiting multidrug resistance (MDR) (Idris and Nadzir, 2023). Conventional antibiotics are losing their edge, prompting an urgent search for novel solutions (Chavada et al., 2023).

One avenue lies in developing new antimicrobial agents targeting novel mechanisms or exploiting weaknesses in resistance pathways. However, the pipeline for new antibiotics is slow and costly (Monserrat-Martinez et al., 2019). Therefore, combinatorial therapies using established and novel agents in strategic combinations are gaining traction (Muteeb et al., 2023). The key lies in synergism, where combined drugs achieve a more significant effect than the sum of their parts. This can enhance efficacy while potentially reducing antibiotic dosage and minimizing side effects (Duarte and Vale, 2022). Research into new agents, optimized combinations, and synergistic interactions is crucial to turn the tide against MDR microorganisms (Kumar et al., 2023). Tackling AMR requires a multi-pronged approach, and these innovative strategies offer a beacon of hope in the fight to preserve the effectiveness of life-saving antimicrobials (OECD et al., 2017).

This Research Topic collects five articles: four are focused on the synergistic effect of different combinations of molecules with antimicrobial activity and conventional antibiotics/antifungals against microorganisms of clinical interest, and one reveals, by using metabolomics, the mechanism of action of combined treatment against *Pseudomonas aeruginosa*.

Chatupheeraphat et al. investigated the potential of peptide K11 (K11) as a novel antibacterial agent and its ability to work with conventional antibiotics to combat drug resistant *Klebsiella pneumoniae* (*K. pneumoniae*). They also explored the antibiofilm activity of this peptide and its stability and ability to induce bacterial resistance. The study found that K11 has potent antibacterial activity against MDR/XDR *K. pneumoniae*, with MIC values ranging from 8-512 mg/mL. When combined with conventional antibiotics, K11 demonstrated a synergistic effect against 53-80% of the tested isolates, particularly when combined with chloramphenicol, meropenem, rifampicin, or ceftazidime. No antagonism was observed in any combination. These findings suggest that K11 has the potential to be used in combination with conventional antibiotics to combat drug-resistant *K. pneumoniae* infections.

The study of Nabavi-Rad et al. suggests that the combination of *Levilactobacillus brevis* (*L. brevis*) and vitamin D3 may have potential as a complementary therapeutic strategy for *Helicobacter pylori* (*H. pylori*) infections, particularly in cases where antibiotic resistance is a concern. The study found that the combination of *L. brevis* and vitamin D3 had anti-inflammatory and anti-oxidative effects against *H*. *pylori* infection in human gastric adenocarcinoma cells (AGS cells) and that the combination of vitamin D3 with live *L. brevis*, pasteurized *L. brevis*, or *L. brevis*-derived membrane vesicles significantly reduced *H. pylori* adhesion to AGS cells. These findings suggest that combining probiotics and vitamin D3 may be a promising approach to reducing *H. pylori*-induced inflammation and preventing *H. pylori* adhesion to gastric epithelial cells. However, further research is needed to fully understand the mechanisms behind this synergistic effect and determine the potential efficacy of this treatment *in vivo*.

In the research paper entitled “Safety and effectiveness of tigecycline combination therapy in renal transplant patients with infection due to carbapenem-resistant gram-negative bacteria,” Wang et al. analyzed the efficacy of using tigecycline combination therapy (with meropenem, imipenem cilastatin, or cefoperazone–sulbactam) on the survival rate and the occurrence of adverse events during the therapeutic regimen in 40 patients. They observed good clinical response in 32 patients (80%) with carbapenem-resistant K. pneumoniae, Acinetobacter baumannii, or Escherichia coli infection. There were no serious adverse events reported among the patients under tigecycline therapy. Nevertheless, increased liver function and pancreatitis precursors were found, compared to their levels before the combinatory treatment. This study positions tigecycline as a good option for combinatory treatment as it resensitizes the resistant bacteria to carbapenem antibiotics.

Using an immunomodulatory drug, Li et al. studied the potential effect of teriflunomide adjuvant in combination with fluconazole as antimicrobial therapy against clinically isolated antibiotic-resistant *Candida albicans* (*C*. *albicans*) This study showed significant synergistic antimicrobial effects using sub-inhibitory concentrations of teriflunomide and fluconazole *in vitro*. The authors also found that this combinatory therapy increased the survival rate of *Galleria mellonella* larvae infected by the clinically isolated *C*. *albicans* and caused a reduction in tissue damage compared with control and monotherapy groups. This work reminds us of the possibility of using non-antifungal drugs as antimicrobial adjuvants capable of causing synergistic interactions and resensitization of antimicrobial agents in resistant microbial strains.

In their study, Yang et al. reminded us that understanding how a combinatorial treatment exerts its effect is as important as finding new working combinations. They analyzed through metabolomic how amikacin (an aminoglycoside) and meropenem (a β-lactam) act in different metabolic pathways when combined compared to their monotherapy effects. This synergistic antibiotic combination, commonly used in clinical therapy, caused modifications in amino acid and nucleotide metabolism, triggering apoptosis signaling, as well as some metabolic pathways like tricarboxylic acid and pentose phosphate pathways, involved in central carbon metabolism pathway, causing a severe disequilibrium in bacterial energy sources, and protection against reactive oxygen species. With this, the authors showed how a combinatorial antibiotic treatment induces significant changes in bacterial metabolism, leading to a faster bacterial death than monotherapy.

We hope this Research Topic provides valuable insight into the synergistic combination treatments and how they represent a pivotal shift in the battle against antibiotic-resistant bacteria. Investing in their development and clinical integration is not merely an option but a critical necessity to protect the foundation of effective antimicrobial therapy.

## Author contributions

JAG-C: Writing – original draft, Writing – review & editing. AL-B: Writing – original draft, Writing – review & editing.
